# Urate dysregulation (hyperuricemia and gout) among people living with HIV: A protocol for a systematic review and meta-analysis

**DOI:** 10.1371/journal.pone.0355217

**Published:** 2026-07-31

**Authors:** Ivaan Pitua, Kim Andrew Otto, Kiragga Denis Lwembawo, Kaweesi Calvin Nantalaga, Maria Gabriella Nampiinga, Michael Ndyomugabe, James Nelson Okema

**Affiliations:** 1 School of Medicine, College of Health Sciences, Makerere University, Kampala, Uganda; 2 Faculty of Medicine, Mbarara University of Science and Technology, Mbarara, Uganda; 3 Faculty of Medicine, Gulu University, Gulu, Uganda; 4 St Mary’s Hospital Lacor, Gulu, Uganda; Guangdong Nephrotic Drug Engineering Technology Research Center, Institute of Consun Co. for Chinese Medicine in Kidney Diseases, CHINA

## Abstract

**Introduction:**

Approximately 39.9 million people are living with HIV worldwide, most in sub-Saharan Africa. Effective antiretroviral therapy has shifted attention to metabolic comorbidities, and emerging evidence links integrase inhibitors such as dolutegravir to elevated serum uric acid, yet the global burden of hyperuricemia and gout in this population is unquantified.

**Methods and analysis:**

This protocol describes a systematic review and meta-analysis of observational studies (cross-sectional, cohort, and case-control) published from inception to May 2026. We will search PubMed/MEDLINE, EMBASE, Web of Science, and CINAHL. Studies involving adults (≥18 years) living with HIV that report the prevalence or incidence of hyperuricemia or gout will be included. Two independent reviewers will screen studies, extract data, and assess risk of bias using the Joanna Briggs Institute Prevalence Critical Appraisal Checklist and the Newcastle-Ottawa Scale (NOS). Primary outcomes are the pooled prevalence of hyperuricemia and gout in PLHIV. Secondary outcomes include risk factors associated with urate dysregulation (ART regimen, Cluster of Differentiation 4 [CD4] count, viral load, metabolic comorbidities, and HIV duration), and gout as an immune reconstitution inflammatory syndrome (IRIS) manifestation. A random-effects meta-analysis using the DerSimonian-Laird method with Freeman-Tukey double-arcsine transformation for prevalence data will be performed in R software. Heterogeneity will be assessed using the I^2^ statistic and Cochran’s Q test. Subgroup analyses will explore variation by ART class (pre-ART, PI, NNRTI, INSTI/dolutegravir era), geographic region, CD4 category, and study quality.

**PROSPERO Registration Number:**

CRD420261360734.

## Introduction

Globally, approximately 39.9 million people were living with HIV (PLHIV) as of 2023, with sub-Saharan Africa bearing over 65% of the world’s HIV burden [1,2]. The sustained scale-up of antiretroviral therapy (ART) has transformed HIV from a near-uniformly fatal infection into a chronic, manageable condition, dramatically extending life expectancy in affected populations [3,4]. However, longer survival has exposed a new epidemiological layer: the rising burden of non-communicable comorbidities driven by persistent immune activation, chronic inflammation, and ART-related metabolic toxicities. Among these, urate dysregulation which involves both hyperuricemia and gout; its principal clinical manifestation, is emerging as an under-recognized but clinically important comorbidity in PLHIV [5–7], consistent with genetic evidence that metabolic traits, including triglycerides and HDL cholesterol, are causally associated with serum uric acid and gout risk [8].

Gout is the most common inflammatory arthritis globally, with a pooled worldwide prevalence of approximately 1–4% in the general adult population and an increasing burden in low- and middle-income countries (LMICs) [9]. It arises from chronic monosodium urate crystal deposition in joints and periarticular tissues, driven primarily by sustained hyperuricemia [10]. In PLHIV, multiple pathophysiological mechanisms converge to elevate serum uric acid: (i) increased purine turnover resulting from accelerated lymphocyte apoptosis and viral replication; (ii) impaired renal urate excretion secondary to HIV-associated nephropathy and ART nephrotoxicity; and (iii) direct uricogenic effects of specific ART agents, particularly ritonavir-boosted protease inhibitors, stavudine, and didanosine [5–7].

More recently, dolutegravir-based regimens, now the WHO-recommended backbone for first-line ART globally, have been associated with hyperuricemia in several sub-Saharan African cohorts, raising important questions about metabolic monitoring protocols in the post-tenofovir era [5–7]. In Uganda, a cross-sectional study at Kiruddu National Referral Hospital identified a hyperuricemia prevalence of 21.3% among PLHIV on predominantly dolutegravir-based regimens [7]. In Ethiopia, multi-centre data documented a prevalence as high as 46.5% among those on dolutegravir-based first-line ART [5]. These rates substantially exceed those reported in HIV-negative general populations in the same regions, signalling a population-specific risk that demands systematic quantification.

Gout in PLHIV presents additional clinical complexity. The only published case-control study specifically designed to measure gout prevalence in an HIV cohort, conducted in Brighton, United Kingdom, identified a point prevalence of 2.2%, substantially higher than the estimated 1.0% in the general adult UK population with hypertension conferring a nearly five-fold increased risk [11]. Ritonavir-boosted protease inhibitor use was established as a risk factor as early as 2005, with an odds ratio of 22 in a retrospective London cohort [12]. Gout has also been described as a rare but distinct manifestation of immune reconstitution inflammatory syndrome (IRIS) following ART initiation, presenting as severe polyarticular flares in the context of Cluster of Differentiation 4 (CD4) recovery [13,14].

Primary studies of urate dysregulation in PLHIV remain few and heterogeneous. Reports from Africa, Asia, and the Americas describe hyperuricemia and gout frequencies ranging from under 1% to over 40%, reflecting differences in study populations, ART regimens, biochemical thresholds, and geographic context [5–7,15–18]. Documented gout rests largely on small case series and individual case reports, the studies rarely stratify by ART class, and few are from sub-Saharan Africa. Despite this growing literature, no systematic review or meta-analysis has pooled these estimates, examined the sources of heterogeneity, or synthesized the risk-factor landscape. This gap undermines the development of clinical monitoring guidelines for urate dysregulation in PLHIV, particularly in sub-Saharan Africa, where dolutegravir-based ART is now universal, rheumatology services are severely limited, and gout is often misdiagnosed as septic or reactive arthritis [19,20]. Because dolutegravir-based regimens are now the World Health Organization preferred first-line therapy globally, an integrase-inhibitor uric acid signal would carry implications well beyond sub-Saharan Africa, including high-income settings where gout prevalence is already high. A preliminary search of PROSPERO, the Cochrane Database of Systematic Reviews and MEDLINE (via PubMed) identified no completed or ongoing systematic review on hyperuricemia or gout among people living with HIV.

Existing reviews of metabolic complications in people living with HIV concentrate on dyslipidemia, insulin resistance, and weight gain and do not synthesize urate outcomes. Urate dysregulation merits separate synthesis because it follows a distinct mechanism involving renal and intestinal urate handling and purine metabolism, carries a specific and modifiable drug signal centred on integrase inhibitors, sits outside standard ART monitoring, and is clinically actionable through an inexpensive assay and treatable disease. A dedicated synthesis is therefore needed to guide monitoring policy.

This protocol describes a systematic review and meta-analysis that will, for the first time, synthesize the global evidence on the prevalence of hyperuricemia and gout in PLHIV, examine how ART class influences urate dysregulation, and identify clinical and demographic correlates that clinicians can use to identify at-risk patients. By framing hyperuricemia and gout under a unified framework of urate dysregulation, we recognize that both conditions exist on a pathophysiological continuum and that their combined synthesis is necessary to fully characterize the burden and to inform rational management strategies. Findings will be particularly relevant to clinical practice in sub-Saharan Africa, where the intersection of HIV, dolutegravir-based ART, and metabolic comorbidities creates a growing, underappreciated rheumatological challenge.

## Methods

This protocol has been drafted in accordance with the Preferred Reporting Items for Systematic Review and Meta-Analysis Protocols (PRISMA-P) 2015 guidelines [21]. The full PRISMA-P checklist is provided in S2 File. The review was registered with PROSPERO (CRD420261360734) before the searches were run.

### Eligibility criteria

#### Study design.

We will include observational studies using cross-sectional, prospective or retrospective cohort, and case-control designs that report quantitative data on the prevalence or incidence of hyperuricemia or gout in PLHIV. Case reports and series with fewer than 10 participants will be excluded. Conference abstracts, reviews, editorials, qualitative studies, and animal studies will also be excluded. There is no restriction on publication year, language, or geographic setting.

#### Participants (P).

Adults aged 18 years and above with documented HIV infection, regardless of ART status, CD4 count, WHO clinical stage, or HIV-1 subtype. Studies that mix paediatric and adult populations will be eligible if adult-specific data can be extracted or if adults constitute ≥80% of the study population. Where a study includes mixed adult and paediatric participants and reports neither the proportion of adults nor adult-specific data, we will contact the corresponding authors with up to two requests. If usable data are not obtained within four weeks, the study will be excluded.

#### Index Condition/Exposure (I).

HIV infection, with or without specific ART regimens. We will specifically characterize the ART class effect by subgrouping studies into: (i) pre-ART/ART-naive era; (ii) protease inhibitor (PI)-based regimens; (iii) non-nucleoside reverse transcriptase inhibitor (NNRTI)-based regimens; and (iv) integrase strand transfer inhibitor (INSTI)-based regimens, particularly dolutegravir. ART exposure will be classified by the regimen the participant was receiving at the time of serum urate measurement (current regimen). Prior ART history will be extracted where reported. Studies reporting only initial or sequential regimens will be recorded as such and examined in a sensitivity analysis, and study populations whose relevant regimen cannot be determined will be excluded from the ART-class stratified analyses.

#### Comparators (C).

For prevalence estimates, comparators are not required but will be recorded where reported: HIV-negative individuals drawn from the same population, or PLHIV on different ART regimens. For risk factor analyses, PLHIV without hyperuricemia or gout will serve as the internal comparator group.

#### Outcomes (O).

Primary outcomes:

- Pooled prevalence of hyperuricemia, defined as serum uric acid >7.0 mg/dL (416 μmol/L) in men or >6.0 mg/dL (357 μmol/L) in women [22,23], or as defined by the individual study with the applied cut-off reported.
- Pooled prevalence of gout, defined by clinical diagnosis, the American College of Rheumatology and the European Alliance of Associations for Rheumatology (ACR/EULAR) classification criteria, or crystal-confirmed diagnosis.

Eligible studies are expected to define hyperuricemia using varying serum urate thresholds. We will record the exact threshold and assay reported in each study, apply standard sex-specific cut-offs (>7.0 mg/dL [416 µmol/L] in men and >6.0 mg/dL [357 µmol/L] in women) as the primary definition, and extract mean or median serum urate where reported so that prevalence can be re-derived at a common threshold. Gout will be accepted as defined by each study (ACR/EULAR criteria, physician diagnosis, or crystal confirmation), recorded by ascertainment method. The effect of these threshold choices is examined in a pre-specified subgroup analysis comparing standard and alternative cut-offs.

Secondary outcomes:

- Risk factors and correlates of hyperuricemia and gout in PLHIV: specific ART agents/classes (protease inhibitors, dolutegravir, stavudine, didanosine), CD4 count, viral load, duration of HIV infection, duration of ART, metabolic comorbidities (hypertension, obesity/overweight, diabetes mellitus, chronic kidney disease, dyslipidemia), and demographic variables (age, sex).
- Gout as an immune reconstitution inflammatory syndrome (IRIS) manifestation: this will be summarized narratively as a descriptive case synthesis rather than pooled, given that the available evidence comprises case reports and small case series.
- Serum uric acid levels (mean/median) in PLHIV versus HIV-negative controls where reported.

#### Study setting.

Any healthcare or community setting globally. Studies from all income settings and geographic regions will be eligible.

### Information sources

The following electronic databases will be searched systematically from inception to May 30, 2026:

1. PubMed/MEDLINE
2. EMBASE (via Elsevier)
3. Web of Science (Core Collection)
4. CINAHL (Cumulative Index to Nursing and Allied Health Literature)

To supplement electronic database searches, we will: (i) hand-search reference lists of all included studies; (ii) review reference lists of relevant narrative reviews and book chapters; (iii) search Google Scholar for the first 100 results of a structured search; and (iv) contact corresponding authors of included studies to identify unpublished or ongoing studies with available data.

### Search strategy

A comprehensive, structured search strategy will be developed for each database in consultation with a trained medical librarian. Search terms will incorporate Medical Subject Headings (MeSH), Emtree terms, and free-text keywords, combined using Boolean operators (AND, OR, NOT). The strategy will combine two conceptual blocks: (i) HIV/PLHIV, and (ii) urate dysregulation (hyperuricemia and gout). To manage the anticipated volume of irrelevant records, the primary search will apply validated “NOT” operators to exclude animal-only studies and non-eligible publication types and screening will be conducted in Covidence, which uses machine learning-assisted reprioritisation after approximately 50–100 records are reviewer-classified. An example PubMed search string is provided below; full database-specific search strings are provided in S1 File.

Example PubMed Search String (See S1 File for all database strings):

(“HIV Infections”[MeSH] OR “HIV”[MeSH] OR “Human Immunodeficiency Virus” OR “PLHIV” OR “people living with HIV” OR “antiretroviral”) AND (“Hyperuricemia”[MeSH] OR “Gout”[MeSH] OR “Arthritis, Gouty”[MeSH] OR “Uric Acid”[MeSH] OR hyperuricemia OR hyperuricaemia OR gout OR “gouty arthritis” OR “uric acid” OR “serum urate” OR “urate dysregulation”)

### Study selection

Search results will be exported to Covidence systematic review software (Veritas Health Innovation, Melbourne, Australia) for automated deduplication. Two independent reviewers (IP, JNO) will screen titles and abstracts against the eligibility criteria. Full-text articles of potentially eligible studies will be retrieved and assessed independently by the same reviewers using a standardized inclusion/exclusion checklist. During extraction, overlapping populations and duplicate publications will be identified by comparing author lists, recruitment sites, and enrolment periods. Where cohorts overlap, the report with the largest sample or most complete outcome data will be retained, and the basis for each decision will be recorded in a table of excluded duplicates. Disagreements at both stages will be resolved through discussion or, if unresolved, adjudication by a third reviewer (KAO). A PRISMA 2020 flow diagram [24] will document the number of records identified, screened, assessed for eligibility, and included in the review, with reasons for exclusion at the full-text stage.

### Data extraction

Data will be extracted by two independent reviewers (IP and JNO) into a standardized Microsoft Excel form, piloted on five randomly selected studies before formal extraction commences. Discrepancies will be resolved by consensus or third-reviewer arbitration (KAO). Where studies report insufficient data, corresponding authors will be contacted by email up to twice over four weeks. Case-control studies will contribute only to risk-factor and association outcomes, such as the association between ART class and hyperuricemia, and will not be included in prevalence pooling. The following data items will be extracted:

#### Study characteristics.

- Author(s), year of publication, country, geographic region (sub-Saharan Africa, South/Southeast Asia, Latin America, Europe/North America, other)
- Study design (cross-sectional, prospective cohort, retrospective cohort, case-control)
- Data collection period, healthcare setting (hospital, community, ART clinic)
- Funding source and conflict of interest disclosures

#### Participant characteristics.

- Sample size, mean/median age and age range, sex distribution
- HIV clinical stage (WHO staging), median CD4 count and range, proportion virally suppressed
- ART status and specific regimen: naive, on PI-based, NNRTI-based, or INSTI-based ART
- Duration of HIV infection and ART exposure, comorbidities (hypertension, diabetes, CKD, obesity)

#### Outcome data.

- Definition of hyperuricemia applied and sex-specific cut-off values (mg/dL or μmol/L)
- Definition of gout (clinical, ACR/EULAR criteria, crystal-confirmed) and diagnostic method
- Number of participants with hyperuricemia and/or gout; crude prevalence estimate with 95% CI
- Mean/median serum uric acid level and standard deviation/IQR
- Risk factors assessed and adjusted/unadjusted odds ratios (OR) or prevalence ratios (PR) with 95% CIs
- Data on gout as IRIS manifestation: time from ART initiation to gout presentation, joints involved, CD4 recovery

### Quality assessment (risk of bias)

Two reviewers will independently assess study quality using validated tools:

- For cross-sectional and cohort studies reporting prevalence data: the Joanna Briggs Institute (JBI) Critical Appraisal Checklist for Studies Reporting Prevalence Data [25], which evaluates nine domains including sampling frame appropriateness, sample size justification, and validity of the measurement instrument.
- For case-control and cohort studies examining risk factors: the Newcastle-Ottawa Scale (NOS) [26], evaluating selection of study groups, comparability, and ascertainment of outcome/exposure.

Studies will be classified as low, moderate, or high risk of bias. High-risk-of-bias studies will be retained in the primary analysis but excluded in sensitivity analyses. Risk of bias will be summarized using traffic-light plots generated in the robvis R package.

### Data synthesis and analysis

#### Primary analysis: Prevalence meta-analysis.

Hyperuricemia and gout will be analysed separately, with independent risk-of-bias assessments, and GRADE certainty ratings. No combined “urate dysregulation” estimate will be generated. Studies reporting both outcomes will contribute independently to each meta-analysis, with cross-outcome interpretation limited to the Discussion. Pooled prevalence estimates for hyperuricemia and for gout will be calculated separately as primary analyses using random-effects models (DerSimonian-Laird method). Prevalence proportions will undergo the Freeman-Tukey double-arcsine transformation prior to pooling to stabilize variance and correct for boundary effects, particularly for studies with very high or very low prevalence. Transformed estimates will be back-transformed for presentation. All analyses will be conducted using the meta and metafor packages in R statistical software (version 4.5.2).

#### Secondary analysis: Risk factors and correlates.

For studies reporting adjusted odds ratios or prevalence ratios for specific risk factors (ART class, CD4 count, hypertension, obesity, CKD, duration of ART), we will pool effect estimates using the generic inverse-variance method within a random-effects framework. Where adjusted estimates are not available, unadjusted estimates will be used and this limitation noted.

#### Assessment of heterogeneity.

Statistical heterogeneity will be quantified using the I^2^ statistic and tested using Cochran’s Q test. I^2^ values will be interpreted as follows: < 25% = low heterogeneity, 25–50% = moderate, 51–75% = substantial, and >75% = considerable heterogeneity. When [27] substantial heterogeneity (I^2^ > 50%) is identified, we will perform pre-specified subgroup analyses and meta-regression to explore sources of heterogeneity.

#### Subgroup analyses.

The following pre-specified subgroup analyses will be conducted:

1. ART class era: Pre-ART/ART-naive vs. PI-based vs. NNRTI-based vs. INSTI (dolutegravir)-based regimens. Where ART regimen is not reported, classification will be inferred from the WHO first-line ART policy in effect during the study period and setting (pre-2013, stavudine-containing; 2013–2018, tenofovir + NNRTI; 2019 onwards, dolutegravir-based). Studies with indeterminate ART class will be excluded from this subgroup analysis but retained in the primary pooled analysis. Sensitivity analyses will compare directly reported versus inferred regimen classifications.
2. Geographic region: sub-Saharan Africa vs. South/Southeast Asia vs. Latin America vs. Europe/North America. A regional subgroup analysis will be conducted only where at least three studies are available per subgroup; otherwise regional estimates will be summarized descriptively.
3. CD4 count category: < 200 (severe immunosuppression); 200–349 (moderate); 350–499 (mild); ≥ 500 cells/μL (immune reconstitution range); and not reported. In addition, CD4 measurements will be further stratified by timing relative to ART initiation: (a) pre-ART CD4 and (b) on-ART CD4 (most recent CD4 while on ART). Where fewer than three studies contribute per stratum, results will be summarised narratively.
4. Study quality: low/moderate risk of bias vs. high risk of bias.
5. Hyperuricemia definition: standard cut-offs (>7 mg/dL male; > 6 mg/dL female) vs. other cut-offs.
6. Setting: high-income vs. low/middle-income countries.

#### Meta-regression.

If a minimum of 10 studies are available for a given outcome, univariable and multivariable random-effects meta-regression will be performed to explore the influence of continuous moderators (mean age, median CD4 count, year of data collection, proportion on INSTI-based ART) on heterogeneity in prevalence estimates.

#### Sensitivity analysis.

Sensitivity analyses will be conducted by: (i) restricting to low/moderate-risk-of-bias studies; (ii) restricting to studies using standard hyperuricemia cut-off values; (iii) restricting to studies with crystal-confirmed gout diagnosis; and (iv) leave-one-out analysis to assess the influence of individual studies on pooled estimates. In the leave-one-out sensitivity analysis, the pooled estimate will be recomputed iteratively with each study omitted in turn. The resulting estimates and heterogeneity statistics will be compared with the overall estimate to identify any single study that disproportionately influences the pooled result.

### Publication bias

If 10 or more studies are included in a meta-analysis for any single outcome, publication bias will be assessed visually using contour-enhanced funnel plots to distinguish true publication bias from heterogeneity-driven asymmetry and statistically using Egger’s linear regression test [28]. Trim-and-fill analysis [29] will be used to estimate the potential impact of unpublished studies on pooled estimates.

### Assessment of certainty of evidence

Two independent reviewers will assess the certainty of the body of evidence for each primary and secondary outcome using the Grading of Recommendations Assessment, Development and Evaluation (GRADE) approach [30]. Evidence certainty will be rated as high, moderate, low, or very low, based on five domains: risk of bias, inconsistency, indirectness, imprecision, and publication bias. GRADEpro GDT software (Evidence Prime, Hamilton, Canada) will be used to generate Summary of Findings tables. For prevalence estimates, we will downgrade certainty based on the risk of bias assessment using the JBI critical appraisal tool.

### Study timeline

The anticipated timeline is as follows:

- PROSPERO registration: April 2026
- Database searching and deduplication: May 2026
- Title/abstract screening: May 2026
- Full-text assessment and data extraction: May 2026
- Risk of bias assessment: May 2026
- Data synthesis and statistical analysis: June 2026
- Manuscript writing and submission: June 2026

### Ethics and dissemination

Ethical approval is not required as the study uses existing published data. Findings will be submitted to a peer-reviewed journal. Results will be presented at regional rheumatology and HIV related conferences to inform clinical practice and ART monitoring guidelines in sub-Saharan Africa.

## Discussion

### Anticipated methodological challenges

Variation in how primary studies define hyperuricemia is the most consequential challenge for this synthesis, because thresholds differ across laboratories and populations and directly determine the prevalence each study reports. We address this by recording the exact threshold and assay in every study, applying standard sex-specific cut-offs as the primary definition, re-deriving prevalence at a common threshold where raw urate values are reported, and testing the effect of threshold choice in a pre-specified subgroup and sensitivity analysis.

Classifying study populations by ART class is complicated by regimen switching over time, since many cohorts include participants with prior exposure to several drug classes. We mitigate this by classifying exposure according to the regimen in use at the time of serum urate measurement, recording prior ART history, examining studies that report only initial or sequential regimens in sensitivity analysis, and excluding from the ART-class analyses any population whose relevant regimen cannot be determined.

The evidence base for sub-Saharan Africa and for longitudinal designs is likely to be thin, which constrains both regional subgroup analysis and causal inference. We will conduct a regional subgroup analysis only where at least three studies are available per subgroup, summarize sparser regions descriptively, and interpret associations cautiously because most contributing studies will be cross-sectional and clinic-based and therefore subject to selection and reporting bias.

Publication bias is a further concern, since hyperuricemia is frequently a secondary endpoint that may be reported selectively. Where ten or more studies contribute to an outcome, we will assess funnel plot asymmetry, apply Egger’s test, and use trim-and-fill to estimate the potential influence of unpublished data.

### Clinical and policy relevance

A confirmed and ART-modifiable burden of urate dysregulation would have one immediately actionable implication, namely the integration of serum uric acid measurement into routine ART follow-up, in the same way that serum creatinine and lipids are already monitored during antiretroviral care [31]. Recognizing gout as a possible manifestation of immune reconstitution inflammatory syndrome carries a second practical implication, since acute polyarticular presentations after ART initiation may be misattributed to septic arthritis and treated with unnecessary antibiotics, hospitalization, and ART interruption in resource-limited settings [13,14]. Whether the hyperuricemia threshold that should prompt intervention in PLHIV differs from the general-population threshold, and whether urate-lowering therapy reduces gout flares, cardiovascular events, and renal progression in this group, are questions this review can frame but not resolve, and they define a clear agenda for prospective comparative studies.

### Strengths and limitations

The principal strength of this review is its comprehensive global scope and its status as the first meta-analytic synthesis of urate dysregulation in PLHIV, with hyperuricemia and gout treated under a single framework so that subgroup analyses by ART class address the most clinically actionable question in the dolutegravir era. The main limitations are anticipated rather than observed and follow from the evidence base. Between-study heterogeneity in definitions, regimens, and settings is expected to be high, most studies are likely to be cross-sectional and clinic-based, and hyperuricemia data are often secondary endpoints with incomplete reporting. The planned harmonization, subgroup, sensitivity, and small-study-effect analyses are designed to characterize and, where possible, reduce the impact of these limitations, and all pooled estimates will be reported with explicit measures of uncertainty.

## Conclusion

Urate dysregulation (hyperuricemia and gout) is an emerging and under-quantified metabolic comorbidity in PLHIV that is mechanistically linked to HIV-related immune activation, viral purine turnover, and direct ART-mediated effects on renal urate handling. The shift to dolutegravir-based ART as the universal first-line regimen in sub-Saharan Africa creates a clear need to characterize the current burden of hyperuricemia in PLHIV and to understand its clinical consequences. This systematic review and meta-analysis will provide the first globally pooled estimates of the prevalence of hyperuricemia and gout in PLHIV, define the ART regimen-specific risk landscape, and generate an evidence base to inform clinical monitoring guidelines and advocacy for rheumatological integration into HIV care. Completion of this review will fill a critical evidence gap and directly inform clinical practice in settings where HIV prevalence and rheumatological need intersect most acutely.

## Supporting information

S1 File Search strings for all databases (PubMed/MEDLINE, EMBASE, Web of Science, CINAHL). (DOCX)

S2 File PRISMA-P 2015 Checklist. (DOCX)
