# Supplementary material for: Urate dysregulation (hyperuricemia and gout) among people living with HIV: A protocol for a systematic review and meta-analysis
Source: PLoS One. 2026 Jul 31;21(7):e0355217. doi: 10.1371/journal.pone.0355217 (PMC13426922; doi:10.1371/journal.pone.0355217)
Supplement: S1 File — (DOCX) [file pone.0355217.s001.docx]

**S1 File**

**Search strings**

**Study-design filtering.** Following peer review, the study-design filter (#3) is not applied to the primary search. The primary combined search for each database is #1 AND #2. This prevents the exclusion of eligible prevalence studies that are not indexed with study-design terms. Study design is instead assessed manually during title and abstract screening. The design-filtered search (#1 AND #2 AND #3, #5) is retained only as a sensitivity comparison, to document the number of records retrieved with and without the design filter.

Four databases were searched: PubMed/MEDLINE, Web of Science (Core Collection), EMBASE (via Elsevier), and CINAHL (via EBSCO). Subject headings were mapped to each platform (MeSH for PubMed, Emtree for EMBASE, and CINAHL Headings), and free-text terms were searched in title and abstract fields.

**PubMed/MEDLINE**

| **No** | **Search strategy** | **Description** |
| --- | --- | --- |
| **#1** | ("HIV Infections"[Mesh] OR "HIV"[Mesh] OR "Acquired Immunodeficiency Syndrome"[Mesh] OR "Anti-HIV Agents"[Mesh] OR "HIV Long-Term Survivors"[Mesh] OR HIV[tiab] OR "HIV-1"[tiab] OR "HIV-2"[tiab] OR "HIV infection"[tiab] OR "HIV infections"[tiab] OR "human immunodeficiency virus"[tiab] OR "human immunodeficiency virus infection"[tiab] OR "human immunodeficiency virus type 1"[tiab] OR "human immunodeficiency virus type 2"[tiab] OR "HIV positive"[tiab] OR "HIV-positive"[tiab] OR "HIV seropositivity"[tiab] OR "HIV seropositive"[tiab] OR "HIV infected"[tiab] OR "HIV-infected"[tiab] OR "HIV patient"[tiab] OR "HIV patients"[tiab] OR "HIV individual"[tiab] OR "HIV individuals"[tiab] OR PLHIV[tiab] OR "people living with HIV"[tiab] OR "person living with HIV"[tiab] OR "persons living with HIV"[tiab] OR "adults living with HIV"[tiab] OR "women living with HIV"[tiab] OR "men living with HIV"[tiab] OR "people with HIV"[tiab] OR "patients with HIV"[tiab] OR AIDS[tiab] OR "acquired immunodeficiency syndrome"[tiab] OR "acquired immune deficiency syndrome"[tiab] OR antiretroviral[tiab] OR "antiretroviral therapy"[tiab] OR "antiretroviral treatment"[tiab] OR "antiretroviral agent"[tiab] OR "antiretroviral agents"[tiab] OR "antiretroviral drug"[tiab] OR "antiretroviral drugs"[tiab] OR ART[tiab] OR HAART[tiab] OR "highly active antiretroviral therapy"[tiab] OR "highly active antiretroviral treatment"[tiab] OR "combination antiretroviral therapy"[tiab] OR "combination antiretroviral treatment"[tiab] OR cART[tiab] OR dolutegravir[tiab] OR efavirenz[tiab] OR tenofovir[tiab] OR "protease inhibitor"[tiab] OR "protease inhibitors"[tiab] OR ritonavir[tiab] OR lopinavir[tiab] OR atazanavir[tiab] OR stavudine[tiab] OR didanosine[tiab] OR "integrase inhibitor"[tiab] OR "integrase inhibitors"[tiab] OR "nucleoside reverse transcriptase inhibitor"[tiab] OR NRTI[tiab] OR NNRTI[tiab] OR INSTI[tiab]) | HIV/PLHIV; population of interest |
| **#2** | ("Hyperuricemia"[Mesh] OR "Gout"[Mesh] OR "Arthritis, Gouty"[Mesh] OR "Uric Acid"[Mesh] OR hyperuricemia[tiab] OR hyperuricaemia[tiab] OR gout[tiab] OR "gouty arthritis"[tiab] OR "uric acid"[tiab] OR "serum urate"[tiab] OR urate[tiab] OR "urate dysregulation"[tiab] OR hyperuricosuria[tiab] OR "monosodium urate"[tiab]) | Hyperuricemia and gout; condition of interest |
| **#3** | ("Cross-Sectional Studies"[Mesh] OR "Cohort Studies"[Mesh] OR "Case-Control Studies"[Mesh] OR "Prospective Studies"[Mesh] OR "Retrospective Studies"[Mesh] OR "Longitudinal Studies"[Mesh] OR "Prevalence"[Mesh] OR "Incidence"[Mesh] OR "Epidemiologic Studies"[Mesh] OR cross-sectional[tiab] OR "cross sectional"[tiab] OR cohort[tiab] OR "cohort study"[tiab] OR "cohort studies"[tiab] OR prospective[tiab] OR "prospective study"[tiab] OR "prospective studies"[tiab] OR retrospective[tiab] OR "retrospective study"[tiab] OR "retrospective studies"[tiab] OR longitudinal[tiab] OR "longitudinal study"[tiab] OR "longitudinal studies"[tiab] OR "case-control"[tiab] OR "case control"[tiab] OR "case-control study"[tiab] OR "case-control studies"[tiab] OR prevalence[tiab] OR incidence[tiab] OR "observational study"[tiab] OR "observational studies"[tiab] OR "descriptive study"[tiab] OR "descriptive studies"[tiab] OR survey[tiab] OR surveys[tiab] OR "follow-up study"[tiab] OR "follow-up studies"[tiab] OR "epidemiological study"[tiab] OR "epidemiological studies"[tiab]) | Observational study designs (applied only in the sensitivity search, not in the primary search) |
| **#4** | #1 AND #2 | HIV with urate dysregulation; primary combined search (no study-design filter) |
| **#5** | #1 AND #2 AND #3 | HIV with urate dysregulation; design-filtered sensitivity search (for comparison only) |

**Web of Science (Core Collection)**

| **No** | **Search strategy** | **Description** |
| --- | --- | --- |
| **#1** | TS=(HIV OR "HIV-1" OR "HIV-2" OR "HIV infection" OR "HIV infections" OR "human immunodeficiency virus" OR "human immunodeficiency virus infection" OR "HIV positive" OR "HIV-positive" OR "HIV seropositivity" OR "HIV seropositive" OR "HIV infected" OR "HIV-infected" OR "HIV patient" OR "HIV patients" OR "HIV individual" OR "HIV individuals" OR PLHIV OR "people living with HIV" OR "person living with HIV" OR "persons living with HIV" OR "adults living with HIV" OR "women living with HIV" OR "men living with HIV" OR "people with HIV" OR "patients with HIV" OR AIDS OR "acquired immunodeficiency syndrome" OR "acquired immune deficiency syndrome" OR antiretroviral OR "antiretroviral therapy" OR "antiretroviral treatment" OR "antiretroviral agent" OR "antiretroviral agents" OR "antiretroviral drug" OR "antiretroviral drugs" OR HAART OR "highly active antiretroviral therapy" OR "combination antiretroviral therapy" OR cART OR dolutegravir OR efavirenz OR tenofovir OR "protease inhibitor" OR "protease inhibitors" OR ritonavir OR lopinavir OR atazanavir OR stavudine OR didanosine OR "integrase inhibitor" OR "integrase inhibitors" OR "nucleoside reverse transcriptase inhibitor" OR NRTI OR NNRTI OR INSTI) | HIV/PLHIV; population of interest |
| **#2** | TS=(hyperuricemia OR hyperuricaemia OR gout OR "gouty arthritis" OR "uric acid" OR "serum urate" OR urate OR "urate dysregulation" OR hyperuricosuria OR "monosodium urate") | Hyperuricemia and gout; condition of interest |
| **#3** | TS=("cross-sectional" OR "cross sectional" OR cohort OR "cohort study" OR "cohort studies" OR prospective OR "prospective study" OR retrospective OR "retrospective study" OR longitudinal OR "longitudinal study" OR "case-control" OR "case control" OR "case-control study" OR prevalence OR incidence OR "observational study" OR "observational studies" OR "descriptive study" OR survey OR surveys OR "follow-up study" OR "epidemiological study") | Observational study designs (applied only in the sensitivity search, not in the primary search) |
| **#4** | #1 AND #2 | HIV with urate dysregulation; primary combined search (no study-design filter) |
| **#5** | #1 AND #2 AND #3 | HIV with urate dysregulation; design-filtered sensitivity search (for comparison only) |

**EMBASE (via Elsevier)**

| **No** | **Search strategy** | **Description** |
| --- | --- | --- |
| **#1** | (HIV OR "HIV-1" OR "HIV-2" OR "HIV infection" OR "HIV infections" OR "human immunodeficiency virus" OR "human immunodeficiency virus infection" OR "HIV positive" OR "HIV-positive" OR "HIV seropositivity" OR "HIV seropositive" OR "HIV infected" OR "HIV-infected" OR "HIV patient" OR "HIV patients" OR "HIV individual" OR "HIV individuals" OR PLHIV OR "people living with HIV" OR "person living with HIV" OR "persons living with HIV" OR "adults living with HIV" OR "women living with HIV" OR "men living with HIV" OR "people with HIV" OR "patients with HIV" OR AIDS OR "acquired immunodeficiency syndrome" OR "acquired immune deficiency syndrome" OR antiretroviral OR "antiretroviral therapy" OR "antiretroviral treatment" OR "antiretroviral agent" OR "antiretroviral agents" OR "antiretroviral drug" OR "antiretroviral drugs" OR HAART OR "highly active antiretroviral therapy" OR "combination antiretroviral therapy" OR cART OR dolutegravir OR efavirenz OR tenofovir OR "protease inhibitor" OR "protease inhibitors" OR ritonavir OR lopinavir OR atazanavir OR stavudine OR didanosine OR "integrase inhibitor" OR "integrase inhibitors" OR "nucleoside reverse transcriptase inhibitor" OR NRTI OR NNRTI OR INSTI) | HIV/PLHIV; population of interest |
| **#2** | ('hyperuricemia'/exp OR 'gout'/exp OR 'uric acid'/exp OR hyperuricemia OR hyperuricaemia OR gout OR "gouty arthritis" OR "uric acid" OR "serum urate" OR urate OR "urate dysregulation") | Hyperuricemia and gout; condition of interest |
| **#3** | ("cross-sectional" OR "cross sectional" OR cohort OR "cohort study" OR "cohort studies" OR prospective OR "prospective study" OR retrospective OR "retrospective study" OR longitudinal OR "longitudinal study" OR "case-control" OR "case control" OR "case-control study" OR prevalence OR incidence OR "observational study" OR "observational studies" OR "descriptive study" OR survey OR surveys OR "follow-up study" OR "epidemiological study") | Observational study designs (applied only in the sensitivity search, not in the primary search) |
| **#4** | #1 AND #2 | HIV with urate dysregulation; primary combined search (no study-design filter) |
| **#5** | #1 AND #2 AND #3 | HIV with urate dysregulation; design-filtered sensitivity search (for comparison only) |

**CINAHL (Cumulative Index to Nursing and Allied Health Literature, via EBSCO)**

| **No** | **Search strategy** | **Description** |
| --- | --- | --- |
| **#1** | ( (MH "HIV Infections+") OR (MH "Acquired Immunodeficiency Syndrome") OR (MH "Antiretroviral Therapy, Highly Active") OR (MH "Antiviral Agents+") OR TI(HIV OR "HIV-1" OR "HIV-2" OR "HIV infection" OR "human immunodeficiency virus" OR "HIV positive" OR "HIV-positive" OR "HIV infected" OR "HIV-infected" OR "HIV patient*" OR PLHIV OR "people living with HIV" OR "person* living with HIV" OR "people with HIV" OR "patients with HIV" OR AIDS OR "acquired immunodeficiency syndrome" OR antiretroviral OR "antiretroviral therapy" OR "antiretroviral treatment" OR "antiretroviral agent*" OR "antiretroviral drug*" OR HAART OR cART OR dolutegravir OR efavirenz OR tenofovir OR "protease inhibitor*" OR ritonavir OR lopinavir OR atazanavir OR stavudine OR didanosine OR "integrase inhibitor*" OR NRTI OR NNRTI OR INSTI) OR AB(HIV OR "human immunodeficiency virus" OR PLHIV OR "people living with HIV" OR "people with HIV" OR antiretroviral OR "antiretroviral therapy" OR HAART OR cART OR dolutegravir OR "protease inhibitor*" OR "integrase inhibitor*") ) | HIV/PLHIV; population of interest |
| **#2** | ( (MH "Hyperuricemia") OR (MH "Gout") OR (MH "Uric Acid") OR TI(hyperuricemia OR hyperuricaemia OR gout OR "gouty arthritis" OR "uric acid" OR "serum urate" OR urate OR "urate dysregulation") OR AB(hyperuricemia OR hyperuricaemia OR gout OR "gouty arthritis" OR "uric acid" OR "serum urate" OR urate OR "urate dysregulation") ) | Hyperuricemia and gout; condition of interest |
| **#3** | ( (MH "Cross Sectional Studies") OR (MH "Prospective Studies") OR (MH "Correlational Studies") OR (MH "Case Control Studies") OR (MH "Prevalence") OR (MH "Incidence") OR (MH "Epidemiological Research") OR TI("cross-sectional" OR "cross sectional" OR cohort OR prospective OR retrospective OR longitudinal OR "case-control" OR "case control" OR prevalence OR incidence OR "observational stud*" OR survey* OR "follow-up stud*" OR "epidemiological stud*") OR AB("cross-sectional" OR cohort OR prospective OR retrospective OR longitudinal OR "case-control" OR prevalence OR incidence OR "observational stud*") ) | Observational study designs (applied only in the sensitivity search, not in the primary search) |
| **#4** | #1 AND #2 | HIV with urate dysregulation; primary combined search (no study-design filter) |
| **#5** | #1 AND #2 AND #3 | HIV with urate dysregulation; design-filtered sensitivity search (for comparison only) |
